# Expression of Concern: HER2 decreases drug sensitivity of ovarian cancer cells via inducing stem cell-like property in a NFκB-dependent way

**DOI:** 10.1042/BSR-2018-0829_EOC

**Published:** 2024-10-07

**Keywords:** HER2, nuclear factor kappaB, Ovarian cancer stem cells

The Editorial Office has been made aware of potential issues surrounding the scientific validity of this paper “HER2 decreases drug sensitivity of ovarian cancer cells via inducing stem cell-like property in an NFκB-dependent way” DOI: 10.1042/BSR20180829, hence has issued an expression of concern to notify readers whilst the Editorial Office investigates. It has been noted that Figure 1E HER2 OE panel seems to appear in a previous publication by Liu et al, 2018 (DOI: 10.1158/0008-5472.can-17-2761), with colour and scalebar modifications. Additionally, the Editorial Office has concerns surrounding similarities in the histological appearance of Figure 6A + and +++ panels. The authors were contacted regarding the concerns, however so far the Editorial Office has not received a response.

